# Evaluating the long-term predictive value of macular thickness fluctuations on diabetic macular oedema response to anti-VEGF treatment

**DOI:** 10.1038/s41433-025-03968-y

**Published:** 2025-09-12

**Authors:** Taseen Alam, Nikhil Das, Christopher Maatouk, Alison Zhao, Rishi P. Singh, Katherine E. Talcott

**Affiliations:** 1 Case Western Reserve University School of Medicine, Cleveland, OH USA; 2 Center for Ophthalmic Bioinformatics, Cole Eye Institute, Cleveland Clinic, Cleveland, OH USA; 3 Department of Ophthalmology and Visual Science, University of Chicago Medicine, Chicago, IL USA; 4 Cleveland Clinic Lerner College of Medicine, Cleveland, OH USA; 5 Cole Eye Institute, Cleveland Clinic Foundation, Cleveland, OH USA; 6 Cleveland Clinic Martin Hospitals, Cleveland Clinic, Stuart, FL USA

**Keywords:** Predictive markers, Retinal diseases

## Abstract

**Objective:**

This study assesses the predictiveness of retinal thickness variability on long-term visual acuity (VA) outcomes in patients with diabetic macular oedema (DMO) undergoing anti-VEGF therapy.

**Design:**

Retrospective chart review at a single institution.

**Participants:**

184 and 138 patients underwent initial treatment for DMO and continued to follow-up at 3 and 5 years, respectively.

**Methods:**

Baseline demographics, past medical, and clinical data were collected through electronic medical record query. Central subfield thickness (CST), choroidal volume (CV), and cube average thickness (CAT) variability over the first year of treatment were calculated using standard deviation, amplitude, and area under the curve. CST, CV, CAT, and best corrected visual acuity (BCVA) were noted at the initial treatment date and 3 and 5 years. CST variability quartiles were compared on baseline, final, and change in BCVA alongside final and change in CST. Multiple linear regression was used to evaluate factors associated with final BCVA at 3 and 5 years while adjusting for confounders.

**Results:**

There is no significant association between larger fluctuations in macular thickness and change in BCVA at 3 and 5 years. Individuals in the highest CST variability quartile had the lowest final BCVA but also the lowest baseline BCVA and no difference in BCVA change. Linear regression revealed that CST variability was not predictive of final BCVA. Baseline BCVA and total number of anti-VEGF injections were predictive of BCVA at 3 years and 5 years respectively.

**Conclusions:**

Macular thickness variability did not predict long-term visual outcomes at 3 and 5 years.

## Introduction

DMO is characterised by the buildup of extracellular fluid in the macula, which occurs when the blood-retina barrier is disrupted alongside a corresponding increase in vascular permeability [1]. Retinal hypoxia leads to vascular endothelial growth factor (VEGF) upregulation with subsequent neovascularization, and these new vessels are increasingly susceptible to leakage of proteins, lipids, and blood [2]. Administration of anti-VEGF agents inhibits VEGF signalling, with the ensuing pathologic cascade providing significant visual and anatomic improvements for DMO patients [2–5].

While DMO patients receiving VEGF treatments have demonstrated significant improvements, there are no established methods for predicting how patients’ vision will specifically respond to anti-VEGF treatments over time [6]. Establishing readily identifiable biomarkers that could predict how patients would respond to anti-VEGF treatments is integral to improving the management of DMO patients going forward [6]. Optical coherence tomography (OCT) enables clinicians to identify several retinal biomarkers, including retinal thickness, intraretinal fluid, and ellipsoid zone integrity [7]. Retinal thickness has come to the forefront as a potential biomarker for predicting response to anti-VEGF treatments in DMO patients.

Investigations of retinal thickness as a biomarker for anti-VEGF response have yielded mixed results [7]. Some investigations have found that reductions in retinal thickness correspond with improved visual outcomes in response to intravitreal anti-VEGF therapy in DMO, but other studies have shown merely a moderate degree of association between retinal thickness and visual outcomes [8–12]. The investigation by the Diabetic Retinopathy Clinical Research Network observed a wide variety of possible visual outcomes for any given central subfield thickness (CST) value, while Bressler et al. found that CST values likely correspond with only a small fraction of visual changes [10–12]. One observed limitation of these studies is that they only use CST values at a single time point, which fails to consider any changes in CST that occur over time. Studies have found that repeated retinal thickness fluctuations may have deleterious effects on photoreceptor functionality, with one demonstrating that repeated mechanical stress on retinal pigment epithelium (RPE) cells leads to RPE damage and VEGF upregulation, thereby inducing angiogenesis and furthering choroidal neovascularization [13]. This link between CST fluctuations and angiogenesis alongside both retinal and choroidal neovascularization makes it a biomarker of potential interest for predicting DMO responses, as these linked processes are key components of DMO pathogenesis.

Given the impact of changes in retinal thickness on photoreceptor cells, it stands to reason that retinal thickness fluctuations may be a valuable indicator of how DMO patients may respond to anti-VEGF therapies. Several studies have evaluated the viability of retinal thickness fluctuation as a biomarker for other macular diseases, finding associations for nAMD and RVO when treated with anti-VEGF treatment [6, 14]. With regards to DMO, Wang et al. found that larger retinal thickness fluctuations over 3 to 12 months are associated with poorer visual outcomes in eyes with DMO treated with anti-VEGF injections [15]. However, no studies to date have evaluated the long-term predictive capabilities of this biomarker. This study aims to assess the relationship between retinal thickness fluctuations and visual outcomes among patients with DMO treated with anti-VEGF injections at three and five years after initiating treatment.

## Methods

### Study design and participants

This study is a retrospective, non-comparative, observational cohort study performed after obtaining approval from the Institutional Review Board. All study-related procedures adhered to the guidelines of good clinical practice (International Conference on Harmonization of Technical Requirements of Pharmaceuticals for Human Use [ICH] E6), applicable FDA regulations, the Health Insurance Portability and Accountability Act, and the Declaration of Helsinki. The electronic medical record was used to identify patients aged 18 years or older with a documented diagnosis of DMO at the Cleveland Clinic Cole Eye Institute from January 2012 to December 2019.

Patients were included if they met the following conditions: initiation of intravitreal anti-VEGF therapy at the Cleveland Clinic for DMO without prior anti-VEGF treatment, OCT scans at baseline, 3, 6, 9, and 12 months (+/− 2 weeks), and follow-up at 3 years or 5 years after the first injection (+/− 3 months). With regards to the treatment protocol, patients received an anti-VEGF injection administration regimen according to the discretion of the clinician, which included medications such as bevacizumab, aflibercept, ranibizumab, or mixed injections. The medications were administered at varying intervals per the discretion of the clinician, with the intention of treating all fluid until dry. To avoid duplication of data and potential biases, in cases where both eyes were eligible only the eye that first received anti-VEGF treatment was selected. A total of 270 patients with a confirmed diagnosis of DMO who were undergoing anti-VEGF treatment at the Cleveland Clinic were identified and screened for the study (Supp Fig. 1). Among them, 184 patients had follow-up data available at the 3-year mark (86 patients were excluded), and 138 patients had follow-up data available at the 5-year mark (132 patients were excluded). Patients were excluded from the study if their eyes had concomitant maculopathies not related to DMO, or if they received steroid injections or focal laser photocoagulation treatment during the study period. Patients who received other ophthalmologic interventions outside of the exclusion criteria (i.e., cataract surgery) during the study period were included in the study.

### Study variables

At the beginning of the study, participant demographics and medical history were gathered, along with details of the treatment administered during the initial visit. BCVA and central subfield thickness (CST) were measured at the baseline, 3-month, 6-month, 9-month, 12-month, 3-year, and 5-year follow-up visits. All BCVA and CST measurements were taken during the same visit. The BCVA measurements were a combination of VA with or without correction, as well as pinhole VA, following the institutional standard protocols. To determine macular thickness parameters, including CST, CV, and CAT (retinal cube average thickness), the Cirrus High-Definition Spectral Domain-OCT Review (V.9.5.1, Carl Zeiss Meditech, Dublin, CA) was utilised [16, 17]. To assess macular thickness variability, the CST standard deviation (SD), CST amplitude, and CST area under the curve (AUC) were measured using the recorded CST values from the end of the loading phase (3 months after treatment initiation) to 12 months.

### Statistical analysis

R Statistical Software (Version 3.6.1, Vienna, Austria) was used to perform statistical analysis. Categorical variables were described in the form of frequencies and percentages, while continuous variables were described using means ± SD. Eyes were stratified into quartiles based on macular thickness variability, with quartile 4 representing the highest degree of variability. Quartiles were used over continuous analysis in order to further visualise the relationship between macular thickness variability measures and visual outcomes, minimise the impact of outliers, and provide a clearer picture of risk strata for potential clinical applications. VA was converted from Snellen to ETDRS via the formula ETDRS  =  85  +  50 × log_10_(Snellen) [16]. Continuous variables such as final BCVA, change in BCVA, final CST, and CST change were compared among quartiles by Kruskal–Wallis multiple comparisons with Dunn’s post-hoc analysis, while categorical variables were compared through chi-square tests. Afterward, multiple linear regression analyses were performed to evaluate whether CST SD, choroidal volume (CV) SD, or cube average thickness (CAT) SD are correlated with final BCVA, while accounting for other factors at baseline. A significance threshold was set at *p* <  0.05.

## Results

Of the 184 patients with 3-year data, the average age was 60.98 ± 11.41 years, 90 (48.9%) were female, and 135 (73.4%) were insulin-dependent. With regards to racial distribution, 131 (71.2%) were White, 40 (21.7%) were Black, and 13 (7.1%) fell into other categories. 105 (57.1%) of the eyes studied were right eyes. In terms of DR staging at baseline, 110 (59.8%) patients had non-proliferative DR (NPDR) and 73 (39.7%) had proliferative DR (PDR). Patients received an average of 8.3 ± 2.47 injections over their first 12 months of treatment. With regards to injection type, 104 (56.5%) received only bevacizumab, 12 (6.5%) received only aflibercept, 1 (0.5%) received only ranibizumab, and 67 (36.4%) received mixed injections. Mean baseline VA was 63.8 ± 15.1 ETDRS letters, and CST at baseline was 407.24 ± 104.0 μm. Baseline CV was 11.96 ± 1.95 mm^3^ and baseline CAT was 330.95 ± 60.19 μm. All the baseline statistics reported for those in the 3-year cohort are listed for the 5-year cohort in Table 1.

**Table 1:** Demographics and Baseline Summary Data.

Variable	DME 3-Year Cohort (*n* = 184)	DME 5-Year Cohort (*n* = 132)
Age (years)	60.98 ± 11.41	60.45 ± 10.87
Gender		
Female	90 (48.9%)	69 (50%)
Male	94 (51.1)	69 (50%)
Race		
White	131 (71.2%)	93 (67.4%)
Black	40 (21.7%)	33 (23.9%)
Other	13 (7.1%)	12 (8.7%)
Eye Laterality		
OD	105 (57.1%)	79 (57.2%)
OS	79 (42.9%)	59 (42.8%)
Diabetic Retinopathy Stage		
NPDR	110 (59.8%)	82 (59.4%)
PDR	73 (39.7%)	55 (39.9%)
Average anti-VEGF injections over 12 months	8.3 ± 2.47	8.2 ± 2.45
Anti-VEGF medication		
Bevacizumab	104 (56.5%)	78 (56.5%)
Aflibercept	12 (6.5%)	8 (5.8%)
Ranibizumab	1 (0.5%)	1 (0.7%)
Mixed (bevacizumab + aflibercept)	67 (36.4%)	51 (37.0%)
Baseline Measurements		
Mean Baseline VA (ETDRS letters)	63.8 ± 15.1	64.5 ± 14.0
Mean Baseline CST (µm)	407.24 ± 104.00	402.04 ± 87.81
Mean Baseline CV (µm)	11.96 ± 1.95	12.92 ± 7.99
Mean Baseline CAT (µm)	330.95 ± 60.19	333.47 ± 38.18

Table includes baseline summary data for the 3-year and 5-year cohort, including age, gender, race, eye laterality, diabetic retinopathy stage, proliferative (PDR) vs. non-proliferative (NPDR) status, VEGF injection number and medication type, as well as baseline BCVA (ETDRS letters), CST (μm), CV (μm), and CAT (μm).

**Fig. 1:**
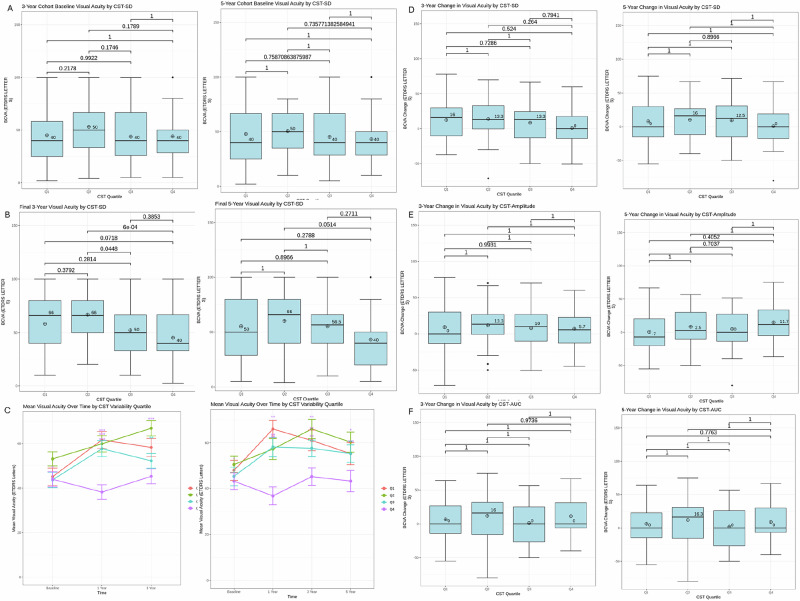
Visual acuity at 3 and 5 years by quartile. Boxplots compare **A** baseline BCVA, **B** final BCVA, and **C** change in BCVA (ETDRS letters) from baseline to 3 and 5 years by each CST variability quartile, as well as **D** 3 year change in visual acuity by CST-SD and **E** 3 year change in visual acuity by CST-Amplitude **F** 3 year change in visual acuity by CST-AUC. Medians are indicated with a line, while mean is indicated by a plus symbol. *P*-values are denoted on the boxplots, with statistical significance at *p* < 0.05 (Kruskal–Wallis multiple comparisons with Dunn’s post-hoc analysis).

**Fig. 2:**
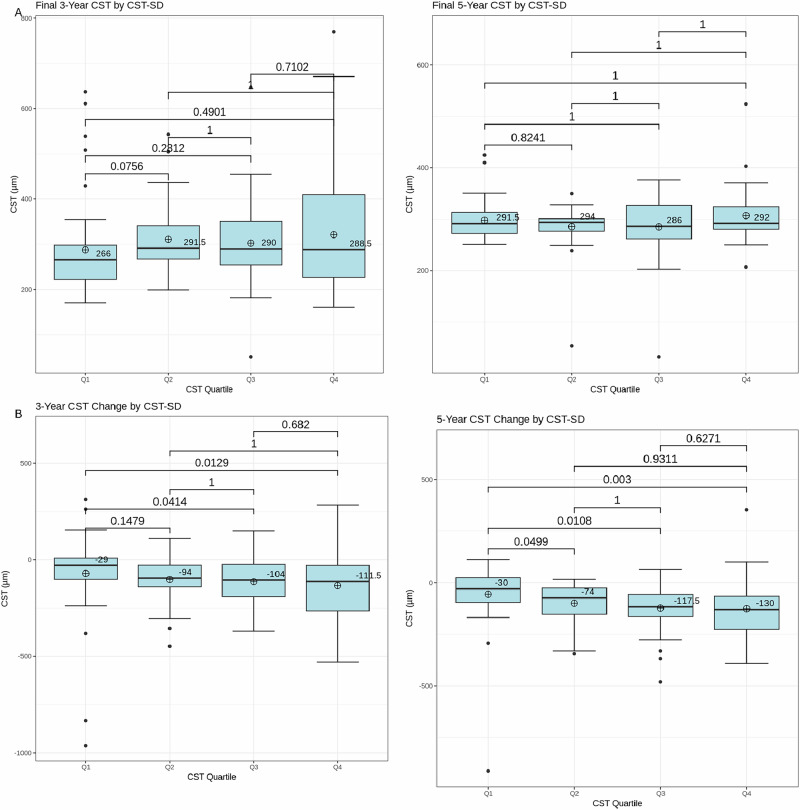
CST at 3 and 5 years by quartile. Boxplots compare (**A**) final CST and (**B**) change in CST by each CST variability quartile. Medians are indicated with a line, while mean is indicated by a plus symbol. *P*-values are denoted on the boxplots, with statistical significance at *p* < 0.05 (Kruskal–Wallis multiple comparisons with Dunn’s post-hoc analysis).

3- and 5- year baseline BCVA by CST-SD, final BCVA by CST-SD, BCVA change by CST-SD, CST-AUC, and CST-amplitude, final CST by CST-SD, and CST change by CST-SD were evaluated for each quartile and depicted in the form of box plots as shown in Figs. 1–2 respectively. Change in VA over time by CST quartile over 3 and 5 years are depicted in Fig. 1C. Means and SDs are shown in Table 2. Comparative analysis of baseline BCVA by CST-SD revealed that none of the quartiles had significantly different baseline BCVAs. Analysis of the final BCVA quartiles demonstrated that the final BCVA was significantly lower in both the third and fourth CST quartiles compared to quartile 2 (*p* < 0.001, p < 0.05) in the 3-year cohort. There were no significant differences in BCVA change among the quartiles over three and five years by CST-SD, CST-AUC, and CST-amplitude, nor were there any differences in the final CST by CST-SD quartiles. The 3-year CST change by CST-SD cohort demonstrated that quartile 1 had a significantly lower CST change than quartiles 3 and 4 (*p* < 0.05, *p* = 0.01). Similarly, for the 5-year cohort, analysis revealed that quartile 1 had a significantly lower change in CST compared to all other quartiles (*p* < 0.05, *p* = 0.01, *p* < 0.01).

**Table 2:** Visual acuity at 3- and 5 years by quartile.

	Quartile 1	Quartile 2	Quartile 3	Quartile 4	*p*-value
Baseline BVA					
3 year	45.1 ± 26.6	53.1 ± 11.1	43.7 ± 23.8	43.9 ± 23.4	0.09
5 year	47.9 ± 26.6	50.6 ± 21.5	45.2 ± 24.0	43.1 ± 21.8	0.47
Final BVA					
3 year	58.3 ± 28.0	66.9 ± 23.1	52.2 ± 22.3	45.3 ± 22.8	**<0.001**
5 year	55.3 ± 28.2	60.3 ± 26.0	55.3 ± 22.8	43.2 ± 27.8	0.06
Change in BVA					
3 year	12.5 ± 29.0	13.9 ± 28.1	8.52 ± 25.2	1.02 ± 26.8	0.21
5 year	7.94 ± 31.6	10.33 ± 30.0	9.50 ± 29.5	0.83 ± 31.7	0.63
Final CST					
3 year	288 ± 102	310 ± 69.5	302 ± 88.6	321 ± 134	0.07
5 year	292 ± 79.4	294 ± 93.4	286 ± 88.1	292 ± 150	0.74
Change in CST					
3 year	−70.6 ± 210	−100 ± 107	−112 ± 113	−133 ± 176	**0.01**
5 year	−56.5 ± 173	−100 ± 92.3	−123 ± 121	−126 ± 153	**<0.01**

Data are presented as mean ± SD. Statistical significance is denoted by *p* < 0.05 and bolded (Kruskal–Wallis multiple comparisons with Dunn’s post-hoc analysis).

Several other variables were evaluated regarding the CST SD quartiles, including baseline CV, baseline CAT, age, number of injections over 12 months, sex, race, eye laterality, presence of PDR, and insulin use. Baseline CV and CAT were found to have statistically significant differences between each quartile. None of the other variables yielded any statistically significant differences.

Multiple linear regression was performed for final BCVA to evaluate which factors had a significant correlation with final BCVA, with proper adjustment for confounders. Baseline BCVA is the only statistically significant predictor of BCVA after 3 and 5 years (Table 3).

**Table 3:** Multiple linear regression evaluating factors associated with final BVA at 3 and 5 years.

	3-year			5-year		
Coefficients	Estimate	SD	*P*-value	Estimate	SD	*P*-value
Intercept	10.46	32.40	0.7492	29.37	23.15	0.2016
CST SD	−0.06	0.05	0.2791	−0.09	0.05	0.1093
Baseline CST	−0.007	0.02	0.7707	0.02	0.02	0.3346
CAT SD	0.02	0.05	0.7423	−0.06	0.09	0.5016
Baseline CAT	−0.01	0.02	0.5454	0.002	0.03	0.9454
CV SD	1.01	1.80	0.5759	−0.06	0.09	0.5016
Baseline CV	−0.49	0.61	0.4199	0.002	0.03	0.9454
CST AUC	0.001	0.005	0.8147	0.005	0.005	0.9916
CST Amplitude	−0.04	0.03	0.2584	−0.06	0.05	0.2204
Baseline ETDRS	0.46	0.02	0.0001**	0.23	0.19	0.00003***
Age	0.03	0.28	0.9078	0.11	0.19	0.5638
Male	7.80	6.01	0.1997	0.78	4.04	0.8468
Other Race	8.11	5.34	0.1309	−3.02	7.83	0.7007
White	4.35	2.95	0.1429	0.42	4.64	0.9285
Left Eye	3.55	2.45	0.1485	1.604	4.004	0.6894
PDR	0.49	2.84	0.8633	−1.98	4.59	0.6667
Insulin Use	0.65	2.89	0.8171	−3.13	4.66	0.5024
Total anti-VEGF injections	0.77	0.51	0.1317	1.74	0.82	0.0865

**≤0.01; ***≤0.001

Statistical significance is denoted by *p* < 0.05 and bolded (multiple linear regression).

## Discussion

This study assessed changes in macular thickness among patients with DMO who received intravitreal anti-VEGF injections, specifically the correlation between macular thickness fluctuations and VA outcomes. The results of the study support the conclusion that there is no significant association between larger fluctuations in macular thickness and visual outcomes at 3 and 5 years. While individuals in the quartile with the highest CST variability had the lowest final BCVA out of all the quartiles, they also had the lowest baseline BCVA, as well as no difference in BCVA change. Performing linear regression analysis revealed that CST variability was not predictive of VA after 3 and 5 years. Other factors that were shown not to be significant predictors of VA at 3 and 5 years include patient gender, age, race, insulin use, and PDR. The only factors that were predictive for final BCVA were baseline BCVA at 3 years and the total number of anti-VEGF injections at 5 years.

Although higher baseline macular thickness can indicate active disease, literature on its relationship with visual outcomes has shown conflicting evidence of macular thickness as a reliable indicator of retinal function [8–12]. Persistent retinal oedema or cytokine activity may lead to dysfunction and damage to neural and glial elements in the retina [17]. While OCT can reveal information about retinal thickness, it does not provide functional information at the cellular level. Consequently, CST inadequately captures molecular-level changes occurring in the retina. Numerous studies have concentrated on exploring the predictive potential of OCT parameters, such as subfoveal choroidal thickness, ellipsoid zone status, subfoveal neuroretinal detachment, and disorganization of the retinal inner layers, among others [18–20]. One study evaluated macular thickness variability as a prognosticator for DMO visual outcomes and found it to be a significant predictor of BCVA at 12 months after initiation of treatment in patients with DMO [15]. Another study analysing the Diabetic Retinopathy Clinical Research Retina Network Protocol T and V found that CST fluctuations serve as a statistically significant predictor of BCVA outcomes at 12 and 24 months [21]. While these studies may have found macular thickness variability to be a reliable predictive variable for visual outcomes at 1–2 years, they did not evaluate change in visual acuity or whether it maintained validity over a longer period of follow-up, such as 3 or 5 years. Moreover, while the second study did note statistical significance after 2 years, this difference in BCVA between patients with different CST fluctuations was reduced compared to 1 year after initiating treatment [21]. This in conjunction with our results supports macular thickness variability as a predictor of short-term outcomes that loses predictive power with time. The mechanism behind our findings merits further investigation; it is possible that other factors like underlying diabetes progression exert greater influence on the rates of angiogenesis and neovascularization with time, thus reducing the predictive power of CST fluctuation as a longitudinal biomarker.

This study has several limitations. Certain factors such as hyperglycaemia were not accounted for and could have potentially influenced the variables studied. Additionally, the presence of diabetic macular ischemia, associated with reduced VA in DMO, might act as another unaccounted-for factor influencing our analysis. The lack of standardised BCVA data, which is a combination of pinhole-corrected, spectacle-corrected, and uncorrected VA, is a common limitation of retrospective studies. Furthermore, due to the retrospective nature of the study, the types of intravitreal anti-VEGF agents patients received were not standardised, with varying treatment intervals and total number of injections over both the 3-year and 5-year periods. Most patients received bevacizumab, which is an off-label treatment for DMO. Even though the type of injection was not shown to be a significant predictive factor amongst our cohort, it is expected to be less effective than aflibercept based on Protocol T outcomes. Moreover, while the number of injections over the first 1-year period was not standardised, it was controlled for by the multiple regression analysis. The average number of injections over the first year was higher than expected based on established literature, but were administered by clinicians per their judgment and best practice guidelines. Additionally, only 1-year data exists in evaluating macular thickness fluctuations upon VEGF injections for DMO patients, with no data from years 1 to 5, which may account for differences seen at 3 and 5 years. It is possible that there were some CST elevations between visits that were not captured on the visit itself but may be captured with daily OCT. Finally, while the multiple linear regression analysis did account for variables which could serve as potential confounders, such as age, number of injections, and baseline BCVA, other confounders may exist that have not been identified or accounted for in our dataset. Therefore, future studies should aim to collect data on a wider variety of patients prospectively, with follow-up to more time points to gain deeper insight. This study investigates the association between macular thickness variability and visual outcomes in patients with DMO receiving anti-VEGF injections after 3–5 years of treatment. The results of this study indicate that there is no statistically significant relationship between macular thickness variability and visual outcomes after 3–5 years of treatment. Future investigations should perform prospective studies on a greater number of patients while collecting data at more time points throughout the study to improve our understanding of macular thickness variability and its clinical implications. Despite its limitations, this study helps provide context to the viability of macular thickness variability as a potential ocular biomarker, demonstrating that it does not have a strong association over 3–5 years of treatment as opposed to shorter time frames. Prospective research into specific morphological characteristics that impact variations in macular thickness, such as alterations in fluid compartments, might pinpoint the precise factors within CST that impact visual results and enhance our prognostic capabilities. Investigating other mechanisms or factors that may come into play over longer time periods may further elucidate additional components affecting longitudinal treatment response to VEGF in patients with DMO.

Supplemental material is available at Eye’s website.

## Summary

### What was known before

- It is known that anti-VEGF therapies are efficacious in treating diabetic macular oedema (DMO).
- There have been studies on various factors that could predict treatment responses to anti-VEGF therapies. One such factor is central subfield thickness (CST).
- CST itself has been shown to not be predictive, but there are conflicting results on whether CST variability is predictive. No study has evaluated this measure over a long period of time.

### What this study adds

- We add a study that evaluates the predictive value of CST variation on anti-VEGF treatment responses in a significant cohort of patients receiving longitudinal care.
- We have found that CST variation does not serve as an accurate predictor for treatment response in patients with DMO over 3 and 5 year periods.

## Supplementary information

Supplemental Figure 1

## Data Availability

The data that support the findings of this study are not openly available due to reasons of patient data confidentiality and are available from the corresponding author upon reasonable request. Data are in controlled access data storage at the Cleveland Clinic Cole Eye Institute.

## References

[1] Virgili G, Parravano M, Evans JR, Gordon I, Lucenteforte E. Anti-vascular endothelial growth factor for diabetic macular oedema: a network meta-analysis. Cochrane Database Syst Rev. 2018;2018:0–0 . 10.1002/14651858.CD007419.pub6 PMC6517135 30325017

[2] Flaxel CJ, Adelman RA, Bailey ST, Fawzi A, Lim JI, Vemulakonda GA, et al. Diabetic retinopathy preferred practice pattern. Ophthalmology. 2020;127:P66–P145. 31757498 10.1016/j.ophtha.2019.09.025

[3] Romero-Aroca P, Baget-Bernaldiz M, Pareja-Rios A, Lopez-Galvez M, Navarro-Gil R, Verges R, et al. Diabetic macular edema pathophysiology: vasogenic versus inflammatory. Exp Diab Res. 2016;2016:1–17. 10.1155/2016/2156273 PMC5059543 27761468

[4] Nguyen QD, Brown DM, Marcus DM, Boyer DS, Patel S, Feiner L, et al. Ranibizumab for diabetic macular edema: results from 2 phase III randomized trials: RISE and RIDE. Ophthalmology. 2012;119:789–801. 22330964 10.1016/j.ophtha.2011.12.039

[5] Korobelnik J-F, Do DV, Schmidt-Erfurth U, Boyer DS, Holz FG, Heier JS, et al. Intravitreal aflibercept for diabetic macular edema. Ophthalmology. 2014;121:2247–54. 25012934 10.1016/j.ophtha.2014.05.006

[6] Chen AX, Greenlee TE, Conti TF, Briskin IN, Singh RP. Fluctuations in macular thickness in patients with retinal vein occlusion treated with anti-vascular endothelial growth factor agents. Ophthalmol Retin. 2020;4:1158–69. 10.1016/j.oret.2020.05.018 32480014

[7] Sheu S-J, Lee Y-Y, Horng Y-H, Lin H-S, Lai W-Y, Tsen C-L. Characteristics of diabetic macular edema on optical coherence tomography may change over time or after treatment. Clin Ophthalmol. 2018;ume 12:1887–93. 10.2147/OPTH.S173956 PMC6165769 30310268

[8] Ou WC, Brown DM, Payne JF, Wykoff CC. Relationship between visual acuity and retinal thickness during anti-vascular endothelial growth factor therapy for retinal diseases. Am J Ophthalmol. 2017;180:8–17. 28549848 10.1016/j.ajo.2017.05.014

[9] Bressler SB. Factors associated with changes in visual acuity and central subfield thickness at 1 year after treatment for diabetic macular edema with ranibizumab. Arch Ophthalmol. 2012;130:1153. 22965591 10.1001/archophthalmol.2012.1107 PMC3543147

[10] Browning DJ, Glassman AR, Aiello LP, Beck RW, Brown DM, Brown DM, et al. Relationship between optical coherence tomography-measured central retinal thickness and visual acuity in diabetic macular edema. Ophthalmology. 2007;114:525–36. 10.1016/j.ophtha.2006.06.052 PMC2585542 17123615

[11] Islam F, Islam F. Retinal thickness and visual acuity in diabetic macular edema: an optical coherence tomography-based study. J College Phys Surg Pak. 2016;26:598–601. 27504553

[12] Bressler NM, Odia I, Maguire MG, Glassman AR, Jampol LM, MacCumber MW, et al. Association between change in visual acuity and change in central subfield thickness during treatment of diabetic macular edema in participants randomized to aflibercept, bevacizumab, or ranibizumab: a post hoc analysis of the protocol t randomized clinical trial. JAMA Ophthalmol. 2019;137:977–985. 10.1001/jamaophthalmol.2019.1963 PMC6604439 31246237

[13] Farjood F, Vargis E. Novel devices for studying acute and chronic mechanical stress in retinal pigment epithelial cells. Lab a Chip. 2018;18:3413–24. 10.1039/c8lc00659h 30328441

[14] Evans LP, Boehme N, Wu S, Burghardt EL. Associations between variation in retinal thickness and visual function. Investigative Ophthalmol Vis Sci. 2020;61:7.

[15] Wang VY, Kuo BL, Chen AW, Chen AX, Chen AX, Chen AX, et al. Fluctuations in macular thickness in patients with diabetic macular oedema treated with anti-vascular endothelial growth factor agents. Eye. 2021;36:1461–1467. 10.1038/s41433-021-01672-1 PMC9232615 34234291

[16] Gregori NZ, Feuer W, Rosenfeld PJ. Novel method for analyzing Snellen visual acuity measurements. Retin- J Retinal Vitreous Dis. 2010;30:1046–50. 10.1097/IAE.0b013e3181d87e04 20559157

[17] Deák GG, Schmidt-Erfurth UM, Jampol LM. Correlation of central retinal thickness and visual acuity in diabetic macular edema. JAMA Ophthalmol. 2018;136:1215. 30193350 10.1001/jamaophthalmol.2018.3848

[18] Campos A, Campos EJ, Carmo A do Caramelo F, Martins J, Martins J, et al. Evaluation of markers of outcome in real-world treatment of diabetic macular edema. Eye Vis (Lond). 2018;5:27. 10.1186/s40662-018-0119-9 PMC6198537 30386806

[19] Sun JK, Lin MM, Lammer J, Prager S, Sarangi R, Silva PS, et al. Disorganization of the retinal inner layers as a predictor of visual acuity in eyes with center-involved diabetic macular edema. JAMA Ophthalmol. 2014;132:1309. 25058813 10.1001/jamaophthalmol.2014.2350

[20] Gerendas BS, Bogunovic H, Sadeghipour A, Schlegl T, Langs G, Waldstein SM, et al. Computational image analysis for prognosis determination in DMO. Vis Res. 2017;139:204–10. 28433753 10.1016/j.visres.2017.03.008

[21] Starr MR, Salabati M, Mahmoudzadeh R, Patel LG, Ammar MJ, Hsu J, et al. Fluctuations in central subfield thickness associated with worse visual outcomes in patients with diabetic macular edema in clinical trial setting. Am J Ophthalmol. 2021;232:90–7. 34283986 10.1016/j.ajo.2021.06.030

