# Supplementary material for: Evaluating the long-term predictive value of macular thickness fluctuations on diabetic macular oedema response to anti-VEGF treatment
Source: Eye (Lond). 2025 Sep 12;39(16):2933–9. doi: 10.1038/s41433-025-03968-y (PMC12583765; doi:10.1038/s41433-025-03968-y)
Supplement: Supplementary file 1 — Supplemental Figure 1 [file 41433_2025_3968_MOESM1_ESM.docx]

**Supplemental Figure 1:** Patient Selection Workflow


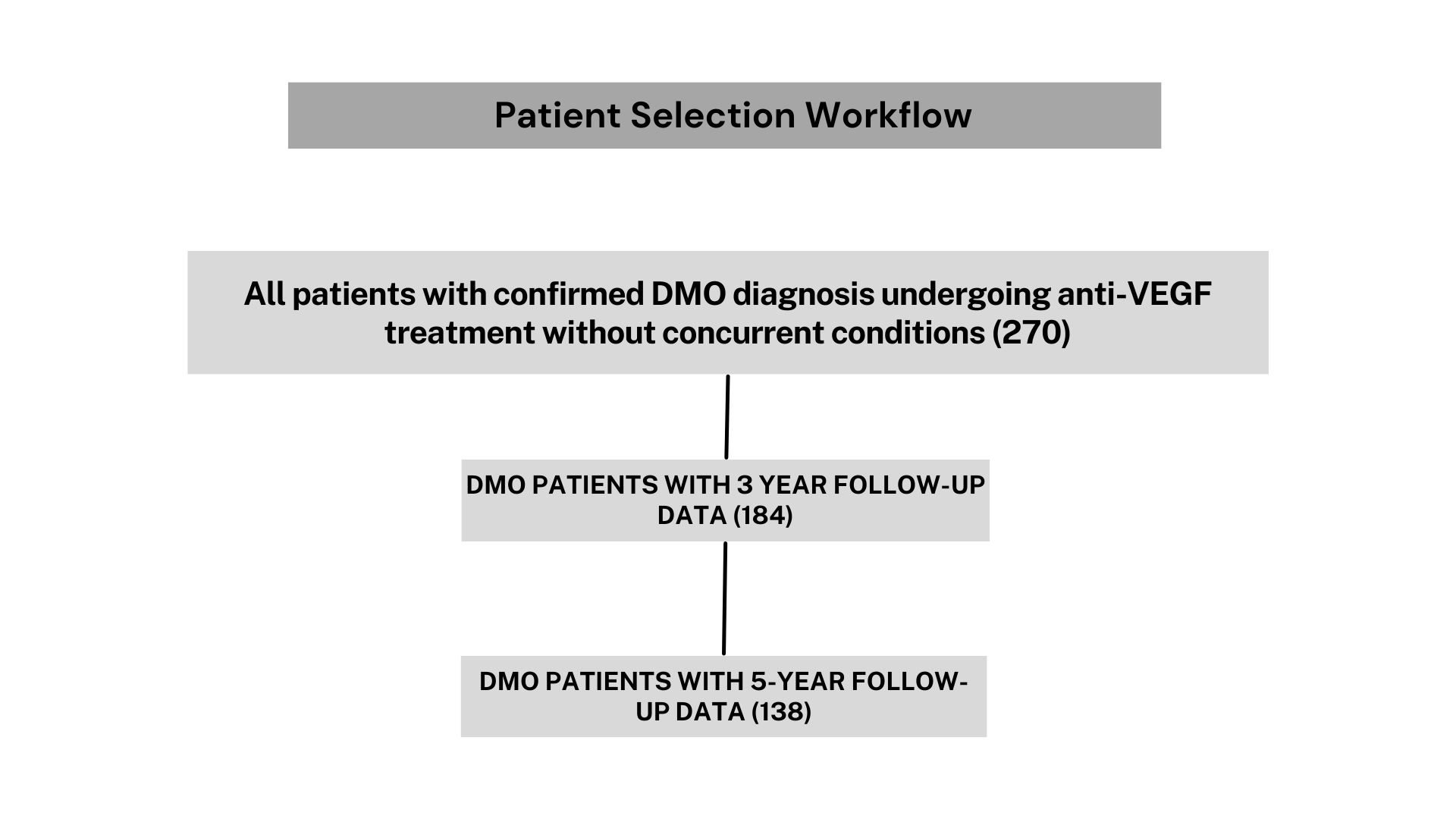


Figure 1: Patient Selection Workflow. 270 patients with a confirmed DME diagnosis undergoing anti-VEGF treatment without concurrent conditions were selected from an electronic record query. From these patients, 184 had 3 year follow-up data and 138 had 5 year follow-up data.
